# My Second Thoughts About Combined Augmentation Mastopexy: A Video Interview With Heather J. Furnas

**DOI:** 10.1093/asjof/ojad078

**Published:** 2023-08-25

**Authors:** Heather J Furnas, Lorne King Rosenfield

In this edition of Second Thoughts on First Thoughts, we share some sage thoughts from another of our esteemed colleagues, Dr Heather Furnas (Video). She harkens from the wonderful legacy of her father, David Furnas, a true icon in our specialty. Now Heather has earned, as they say, her own stripes, commanding a stellar private practice with her husband Paco Canales.

In this video interview, Dr Furnas shares one of her favorite “aha” surgical moments: ultimately enjoying a more reliable and safe strategy for the combined augmentation mastopexy approach. And to round out the segment, you will hear some wise sentiments about practice, patients, and life as passed on by her father. Thanks for listening!

## Supplementary Material

ojad078_Supplementary_Data

